# 2663. Comprehensive at-home Sexually Transmitted and Blood Borne Infection (STBBI) Testing Program: A Pilot Initiative

**DOI:** 10.1093/ofid/ofad500.2274

**Published:** 2023-11-27

**Authors:** Brandon L Christensen, Caley B Shukalek, Grace (Juyoung) Kwon, Nolan E Hill, Deirdre L Church, Christopher T Naugler, John Kim, Ranjani Somayaji

**Affiliations:** University of Calgary, Calgary, Alberta, Canada; University of Calgary, Calgary, Alberta, Canada; University of Calgary, Calgary, Alberta, Canada; Centre for Sexuality, Calgary, Alberta, Canada; University of Calgary, Calgary, Alberta, Canada; University of Calgary, Calgary, Alberta, Canada; National Microbiology Laboratory, Public Health Agency of Canada, Winnipeg, Manitoba, Canada; University of Calgary, Calgary, Alberta, Canada

## Abstract

**Background:**

Sexually transmitted and blood borne infection (STBBIs) remain a growing concern in North America with rates increasing dramatically since 2000. Although self-testing has been demonstrated to be valid and acceptable, no fully remote STBBI testing programs are available in Canada. To evaluate feasibility and acceptability of at-home STBBI testing, we implemented a fully remote, web-based platform of risk-responsive, non-invasive STBBI diagnostics in a large Canadian health zone.

**Methods:**

A web-based risk assessment and intake form was developed using RedCap software through a community-facing website (athomestitesting.ca) in partnership with a local sexual health centre. Demographic, sexual practices, and medical history were collected from interested participants through the platform. Diagnostic kits inclusive of testing for gonorrhea/chlamydia (urine and self-swabs of vagina/pharyngeal/rectal) and syphilis/HIV/Hepatitis C (dried blood spot) were mailed to participants based on their survey responses, along with instructions and supplies for collection and return. Outcomes to be assessed include feasibility and acceptability of the web-based platform, testing and positivity rates for STIs, and time to testing and treatment (for positive cases).

**Results:**

In the first month to date with limited launch, eleven participants completed the intake survey and were mailed testing kits through the platform. Of the completed testing, no new STIs were identified. The median age of the cohort was 38 years old (IQR 32, 41) and six identified as men, four as women and one non-binary. Regarding gender identity, seven identified as gay/queer/bisexual and three as straight/heterosexual. In a follow-up survey, all participants to date stated reported satisfaction or extremely satisfaction with the online testing.

**Conclusion:**

The platform model demonstrates feasibility in the early pilot phase with acceptability based on participant responses. Next steps include expanded launch of the platform and assessment of testing rates and feasibility of a web-based model for STI diagnostics.

**Disclosures:**

**Caley B. Shukalek, MD, MSc, MPH**, Gilead Sciences: Advisor/Consultant|Gilead Sciences: Grant/Research Support|Gilead Sciences: Honoraria|Merck Canada: Grant/Research Support|Merck Canada: Honoraria|PurposeMed Inc: Advisor/Consultant|PurposeMed Inc: Ownership Interest **Ranjani Somayaji, M.D.**, CF Foundation: Grant/Research Support|CIHR: Grant/Research Support|Oncovir: Data and Safety Monitoring Board Fees|Vertex Pharmaceuticals: Advisor/Consultant|Vertex Pharmaceuticals: Grant/Research Support|Vertex Pharmaceuticals: Honoraria

